# Parental Knowledge, Attitudes, and Behaviours towards Human Papillomavirus Vaccination for Their Children: A Systematic Review from 2001 to 2011

**DOI:** 10.1155/2012/921236

**Published:** 2011-10-02

**Authors:** Kristina Trim, Naushin Nagji, Laurie Elit, Katherine Roy

**Affiliations:** ^1^Bachelor of Health Sciences Program, Faculty of Health Sciences, McMaster University, Hamilton, ON, Canada L8S 4L8; ^2^Ontario Cervical Screening Program, Cancer Care Ontario and Division of Gynecologic Oncology, Juravinski Cancer Centre, Hamilton, ON, Canada L8V 5C2; ^3^Morden Street Research Services, Hamilton, ON, Canada L8S 4S3

## Abstract

*Objectives*. A systematic review of parental surveys about HPV and/or child HPV vaccination to understand parental knowledge, attitudes, and behaviour before and after FDA approval of the quadrivalent HPV vaccine and the bivalent HPV vaccine. *Search Strategy*. Searches were conducted using electronic databases limited to published studies between 2001 and 2011. *Findings*. The percentage of parents who heard about HPV rose over time (from 60% in 2005 to 93% in 2009), as did their appreciation for the HPV infection and cervical cancer link (from 70% in 2003 to 91% in 2011). During the FDA approval, there was a stronger vaccine awareness but it has waned. The same pattern is seen with parents whose children received the HPV vaccine (peak at 84% in 2010 and now 36% in 2011) or the intention to vaccinate (peak at 80% in 2008 and now 41% in 2011). *Conclusions*. Parents had safety concerns and wanted more information their physician from to recommend and to confidently HPV vaccinate their children.

## 1. Background

Human papillomavirus (HPV) is the most common sexually transmitted infection in the world and is an established causative agent for cervical, anal, and penile cancers, as well as genital warts in both men and women [1, 2]. It is estimated that 75% of Canadians will experience an HPV infection at least once in their lifetime, with the highest rates of infection occurring in individuals under the age of 25 [3]. In June 2006, the US Food and Drug Administration (FDA) approved the quadrivalent vaccine for use in the prevention of HPV strains 6, 11, 16, and 18, which are associated with 70% of cervical cancer and 90% of genital warts cases [4, 5]. In October 2009, the bivalent HPV vaccine was approved by the FDA for the prevention of HPV strains 16 and 18 which are associated with 70% of cervical cancer cases [4, 6]. Unlike the quadrivalent vaccine, the bivalent HPV vaccine does not protect against strains of HPV that cause genital warts [6]. Both vaccines are administered in three doses over a period of six months. 

As a result of the approval of the HPV vaccines, recent health policy discussions have introduced the idea of adjusting the age of initial PAP smears from 18 years old (or with sexual debut) to 21 or 22 years old (or with sexual debut) [7]. Additionally, a move away from PAP smears toward HPV viral testing for women over 30 with a concurrent decrease in the frequency of PAP smear testing from annually to every 3 to 5 years has been proposed [7]. These health policy shifts are rooted in the success of the HPV vaccines to guard against cervical cancer. This success is, necessarily, dependent on successful vaccine uptake. 

Currently, policy is modelled on an 80% uptake by young women, which means when combined with vaccination, reducing the frequency of testing and increasing the age of initial PAP smear would be part of an efficient plan to reduce cervical cancer. However, actual uptake of the vaccines is relatively low and not consistent in all areas that the vaccine is offered. For example, in the province of Quebec where there is a passive consent strategy to school immunizations (i.e., parental consent must be explicitly withdrawn in a note to the school), there is an 80% vaccine uptake for grade-8 girls [8, 9]. However, in the province of Ontario, where the school-based immunization program has an active consent strategy (i.e., parental consent is explicitly given in a note to the school), the vaccine uptake rate for grade 8-girls is 50% [8, 9]. By comparison, the acceptance of the hepatitis B vaccine was accepted without difficulty yet both aim at preventing disease that is sexually acquired. The acceptance of the hepatitis B vaccine for grade-7 students in Ontario was 79.8% (range 65.2% to 95.2%) and in Quebec the acceptance for grade-8 students was between 85 and 95% [8, 9]. The hepatitis vaccine is offered to both boys and girls and is marketed to prevent liver disease and liver cancer, which is relatively rare in the developed world compared to cervical cancer. Suggested reasons for a low vaccine uptake rate range from low knowledge levels regarding HPV and the HPV vaccine, to cost, to a perceived low efficacy of the vaccine. Recent literature has examined these possible factors as they relate to adolescent attitudes towards HPV vaccination [7]. However, given that the vaccines are targeted towards males and females in the 9- to 26-age group, with emphasis placed on ages 11 and 12 in order to promote inoculation prior to sexual debut [10], a key factor in the implementation of HPV vaccines is the extent to which parents accept HPV vaccination for their children. In order to fully understand the issues surrounding HPV vaccine uptake, parental attitudes towards the vaccine must be examined.

## 2. Objectives

The purpose of this systematic paper is to compare the findings of previous studies that have examined parental knowledge, attitudes, and behaviours towards the HPV vaccine. Particular emphasis will be placed on changes within parental knowledge, attitudes, and behaviour following the availability of the HPV vaccines. Beyond identifying trends in uptake, the paper will also focus on factors that affect parental intentions to vaccinate their children against HPV. Based on previous studies regarding vaccination, these factors may include parental knowledge regarding cervical cancer and STIs (i.e., genital warts); perceived risk and severity of cervical cancer and genital warts; attitudes towards vaccines in general; issues concerning increase in sexual activity or promiscuity; availability of health insurance to cover vaccine costs. Additionally, a preliminary analysis of parental attitudes towards STI-prevention interventions versus anticancer interventions will be conducted to determine the policy implications of the two vaccines; one of which is part of an anticancer strategy as well as STI (genital warts) prevention and one of which only targets cervical cancer prevention. 

## 3. Method

### 3.1. Search Strategy

Prior to conducting the literature search, a librarian was consulted for assistance in building a comprehensive search strategy. Relevant research studies were located through an extensive search of the electronic databases PubMed, Ovid MEDLINE, Embase, and Cumulative Index of Nursing and Allied Health Literature (CINAHL). Searches were limited to only those studies which were published between 2001 and 2011 in an effort to obtain the most recent articles regarding parental knowledge, attitudes, and behaviour before and after FDA approval of the quadrivalent HPV vaccine and the bivalent HPV vaccine.

A preliminary hand search of the literature was completed in order to identify appropriate keywords and medical subject heading (MESH) terms. The terms that were selected to be used in this paper were “HPV”, “parent” and “vaccine,” with “parent-child relations,” “papillomavirus vaccine,” “parent or child parent relation,” and “wart virus vaccine” often being utilized as synonyms. Search terms were combined using the operators “AND” and “OR” to ensure that all relevant articles were located.

### 3.2. Study Selection

**Figure 1:**
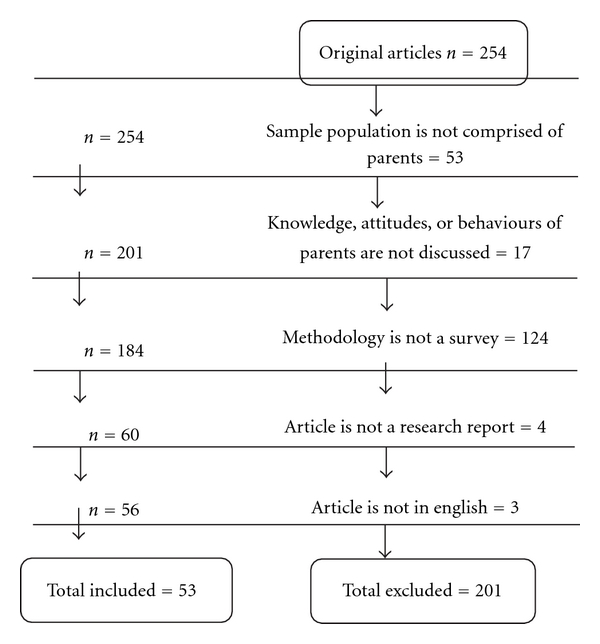
Inclusion and exclusion process.

A total of 325 articles were identified: 304 from the initial search strategy and an additional 21 articles were gathered from the hand search of the literature. Following the removal of duplicates (*n* = 71), 254 articles were screened for inclusion in the paper (Figure 1). Inclusion criteria included (a) the study was about parents and their attitudes towards HPV and/or HPV vaccination; (b) the report had cross-sectional data about the parent's knowledge, attitudes, or beliefs about HPV that were not previously influenced by the research team with an intervention. Exclusion criteria included (a) sample population was not comprised of parents; (b) knowledge, attitudes, and/or behaviours of parents not discussed in results; (c) methodology of study did not include survey; (d) article was not based on original research (i.e., the study was a literature review); (e) the full article was not available in English. The results of the study selection process are shown in Figure 1.

### 3.3. Data Collection and Abstraction

The data abstraction form was created and was pilot tested on five articles. Once the remaining modifications were made to the abstraction form, three coders extracted the data based on the coding information provided on the form. The data was then entered into SPSS for analysis, with information being transformed into percentages where possible. Initially, five outcomes that correspond to parental knowledge, attitudes, or behaviours were recorded. These five outcomes were that the parents had heard of HPV, heard of the HPV vaccine, knowledge of association between HPV and cervical cancer, an intention to vaccinate their child, and vaccinated their child(ren) with one or more doses of the vaccine. Following this initial analysis, an analysis of factors affecting parental attitudes toward the vaccines was conducted.

## 4. Results

### 4.1. Characteristics of Study Samples

**Figure 2:**
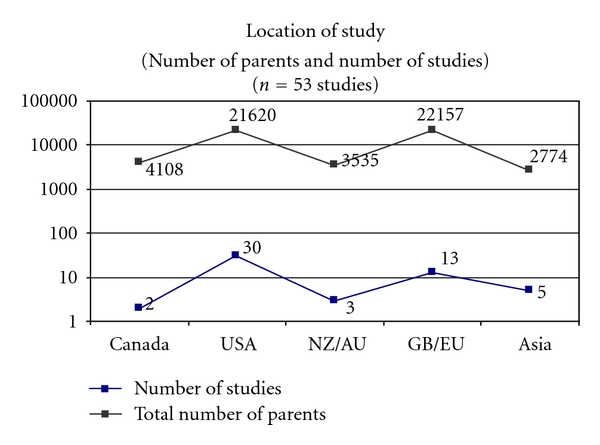
Location of study.

**Figure 3:**
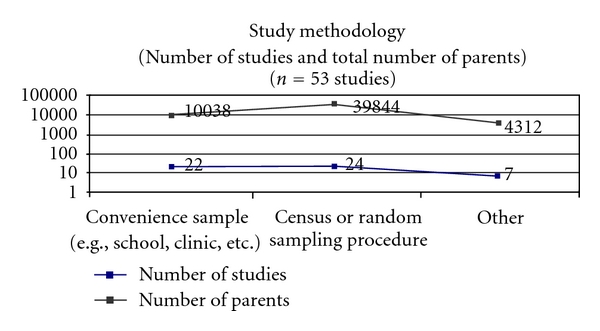
Study methodology.

**Figure 4:**
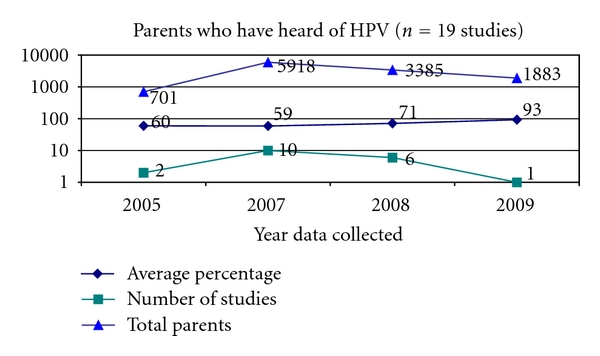
Heard of HPV.

The literature search resulted in 53 studies that met inclusion criteria and were included in this systematic paper [11–63]. All included studies have been listed by publication date, research question, and focus in Figure 4. Publication dates were between the years of 2004 and 2011 with the majority of the studies being published in 2009 (28.3%) and 2010 (34.0%). Surveys were administered to parents in 2007 or earlier in 60.4% of the studies. The majority of the studies were conducted in North America (USA: 56.6%; Canada: 3.8%), however, the European Union (24.5%), Asia (9.4%), and New Zealand or Australia (5.7%) were also represented. Figure 2 highlights the geographic representation of the sample. Forty-one percent of studies were conducted in a school or medical setting, while 45.3% used some form of population-based sampling (e.g., census or government data, random digit dialling, or an existing longitudinal study), and 13.2% used other sampling procedures. These “other” sampling procedures included pretest data from an educational HPV intervention or mixed methods.  Figure 3 demonstrates the methodology of the studies in the sample.

The total number of parents included in this study was 54,194 with a median study sample size of 506, and a mean study sample size of 1,022 (SD = 2,099). Six studies only reported “parents” and did not differentiate between mothers and fathers. Twenty-three studies (43%) reported mothers' responses only. Of those studies that reported both mothers' and fathers' attitudes, the majority of respondents were mothers with the average sample size of mothers at 82.3% (minimum: 47.7% and maximum: 95.1%).

### 4.2. Framework for Analyses

Studies posed a wide variety of research questions; in order to simplify analyses, each study's central question and results were grouped according to whether they were concerned with parental knowledge of HPV, parental behaviours toward the HPV vaccines, parental intent to vaccinate their children against HPV (attitudes), or a combination of the three factors (Table 1). 

Of the 53 studies included, 73.6% (39/53) attempted to gauge parental knowledge of HPV and the HPV vaccine. This ranged from whether parents were aware of HPV to whether parents could correctly identify HPV as the causative agent of cervical cancer. Thirty-eight percent (20/53) of studies focused on parental behaviour (i.e., whether parents had already inoculated their child or children against HPV with either of the two vaccines available). Finally, 92.5% (49/53) of studies focused on parental attitudes toward the vaccine (whether parents intend to vaccinate their children against HPV). Many studies also focused on factors affecting parental attitudes and behaviour regarding the HPV vaccine. These factors included perceived vaccine efficacy; vaccine safety; perceived threat of HPV. They will be discussed more thoroughly in the section regarding factors and barriers.

### 4.3. Knowledge Trends

Three primary knowledge questions were examined: whether parents had heard of HPV, whether they had heard of the HPV vaccine, and whether they could correctly identify the relationship between HPV and cervical cancer. Of the 53 studies, 19 studies (36%) asked parents whether they had heard of HPV prior to being included in the study (Figure 4). 

**Figure 5:**
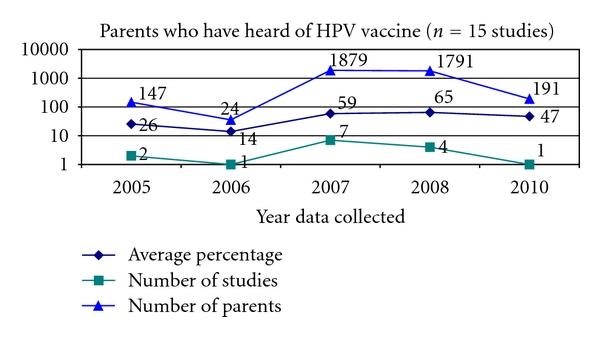
Heard of HPV vaccines.

Parental awareness of HPV increased in 2008 and 2009. Of the 53 studies, 15 studies (28%) asked parents whether they had heard of the HPV vaccines prior to being included in the study (Figure 5). 

**Figure 6:**
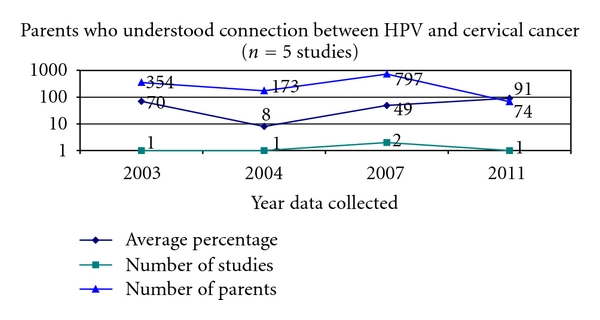
Cervical cancer and HPV.

Parental awareness of the HPV vaccine spiked in 2007 with a mean percentage of 59% compared to 14% in 2006. Awareness continued to climb to 65% in 2008 and dropped off slightly to 47% by 2010. These years are of particular interest since they mark the introduction and availability of the quadrivalent HPV vaccine and the bivalent HPV vaccine. Of the 53 studies, 5 studies (9%) asked parents if they could identify the relationship between cervical cancer and HPV (Figure 6).

It is important to note that only 5 studies asked parents to make the connection between HPV and cervical cancer. In the study in which data were collected most recently (2011), an average of 74% of parents could correctly identify the relationship between HPV and cervical cancer. With only 5 studies examining parental knowledge of the relationship between HPV and cervical cancer, it is difficult to make any connections between knowledge and the introduction and availability of the HPV vaccine.

### 4.4. Behaviour Trends

**Figure 7:**
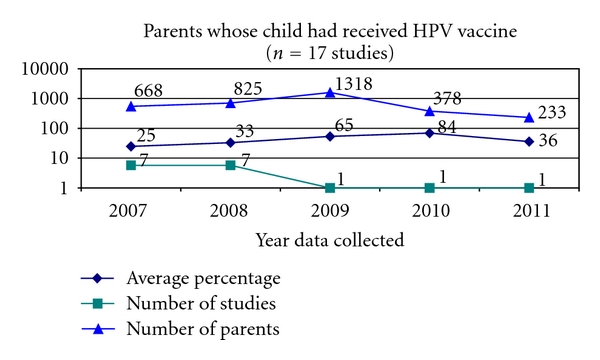
Received HPV vaccine.

Of the 53 studies, 17 studies (32%) asked parents whether their child or children had already been vaccinated against HPV (Figure 7).

Following the availability of the quadrivalent HPV vaccine in 2007, studies began asking whether parents had already vaccinated their children against HPV. The highest percentages of parents who had vaccinated their children against HPV occurred in 2009 and 2010. This is following the introduction and availability of the bivalent HPV vaccine in 2009.

### 4.5. Attitude Trends

**Figure 8:**
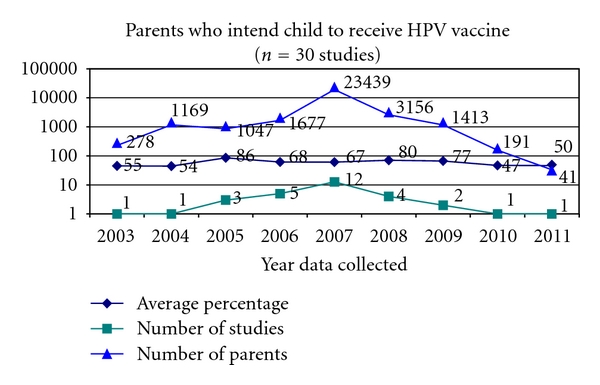
Intent to inoculate against HPV.

Of the 53 studies included, 30 studies (57%) asked parents whether they intend to vaccinate their child or children against HPV (Figure 8). 

The highest percentage of parents who intend to vaccinate their children (86%) occurs in studies where the data were collected in 2005, prior to the release of the first HPV vaccine. Intent increases in 2008 to 80% of parents from 67% in 2007 and then gradually decreases in 2009, 2010, and 2011.

All three knowledge components have increased from pre-2007 studies to post-2007 studies. While levels of uptake pre-2007 and post-2007 cannot be compared, intent to vaccinate has decreased from pre-2007 to post-2007 (Table 2).

### 4.6. Factors Affecting Parental Decision to Vaccinate

Of the 53 studies included, 81% made some mentioning of examining barriers to parental intent to vaccinate. Parental experiences and demographic characteristics were too mixed to show any clear pattern within the 53 studies. Cost factors were also mentioned, but were difficult to compare across studies.

### 4.7. Knowledge

**Parents Concerned about the Safety of the HPV Vaccine**

In 20 studies (37%), parents expressed concerns about vaccine safety and the potential side effects of the HPV vaccine [13, 15, 20, 23, 30, 32, 33, 35, 37, 39, 41, 42, 46–48, 52–54, 60, 61].

**Parents Wanted More Information about the Vaccine to Make an Informed Decision as to Whether They Should Vaccinate Their Child with the HPV Vaccine**

In 13 studies (25%), parents needed more information about HPV vaccination [17, 30, 34, 41, 42, 46–48, 51, 52, 54, 60, 62].

### 4.8. Attitudes

**Parents Who Were Concerned about the Potential Risk of Cancer Were More Likely to Vaccinate for HPV**

In 16 studies (30%), parents mentioned a concern about cancer risk as increasing the likelihood of HPV vaccination. Parents who believed it was likely that their daughters might contract HPV [13, 15, 18, 31–33, 50, 52], develop cervical or penile cancer [13, 18, 20, 30, 31, 39, 47, 50, 53, 60, 62, 63] or genital warts [13, 18, 47, 62] were more likely to vaccinate their daughters.

**Parents Agreed That Children Should Be Older and Sexually Active to Receive the Vaccine**

There were 10 studies (19%) that mentioned the child's age affecting the parents' decision to vaccinate. Parents were less likely to vaccinate their children if they believed their children were too young or not sexually active [34, 40, 44, 48]. Some studies indicated that parents were more likely to vaccinate their children if they were sexually active [23] or older [32, 34, 53–55, 57].

**Mixed Opinions About Parental Concerns for Increased or More Risky Sexual Activity If Child Is Vaccinated**

Parents were not concerned that their children would become sexually active if they were given the HPV vaccine in 13 studies (25%) [12, 14, 17, 28, 32–34, 39, 42, 46, 51, 60, 62], while in six studies (13%), parents expressed concerns that the HPV vaccine might encourage earlier sexual initiation, or more risky sexual behaviours in their children [15, 23, 43, 48–50, 61].

### 4.9. Behaviours

**Parents Looked to Their Physicians to Recommend the HPV Vaccine**

In 17 studies (32%), parents indicated that having their doctors recommend the vaccine increased the likelihood of HPV vaccination [17, 19, 24, 28, 30–35, 41, 47, 51–53, 60, 62].

**Parental Attitudes towards Vaccines Generally Indicated Whether They Were in Favour of Vaccinating Their Children for HPV**

Parents who had previously vaccinated their children against meningitis [19] or had a general belief in the efficacy of vaccines [15, 24, 25, 32, 45, 48–50, 62] were more likely to vaccinate (10 studies or 19%). Parents who had refused previous vaccines for their children [11, 18, 45, 47, 63] and had concerns about too many vaccinations [45, 60] were less likely to vaccinate (6 studies or 11%).

## 5. Discussion

This paper reviews the parental knowledge, attitudes, and behaviours toward having their daughters and sons vaccinated against cervical cancer. The parents in these studies were largely from a high resource background. The percentage of parents that participated in these surveys who had heard about HPV clearly rose over time (from 60% in 2005 to 93% in 2009). Parents' appreciation for the link between HPV infection and cervical cancer did rise (70% in 2003 to 91% in 2011). During the era of FDA approval of the vaccines, there appeared to be stronger awareness of the vaccines and this has waned with time. This same pattern is seen with the percentage of parents whose children had received the HPV vaccine (high of 84% in 2010 and now 36% in 2011). Unfortunately, this pattern is also seen with the intention to have a child vaccinated against HPV (peak at 80% in 2008 and now 41% in 2011). 

In terms of barriers against the vaccine, parents still have safety and side-effect questions and they want more information. Parents view the vaccine like the oral contraceptive pill; it is best to invest in it only when you become at risk (i.e., you are sexually active). Parents who have high cancer worries and receive strong messages about HPV risks are more likely to advocate for the HPV vaccine. Parents look to their physicians to recommend the vaccine. 

The strengths of this study are that it involves information gathered from a large number of parents from several countries. It shows trends in knowledge, attitudes, and behaviours over a time period just preceding the FDA approval of the vaccine; during the approval phase when there were extensive educational campaigns both by the pharmaceutical companies, professional societies, and media, after the FDA approval. The limitations of this study was an inability to validate parental responses, for example, determining how many parents had their child vaccinated with at least one dose of the vaccine. 

It will be interesting to see if there are changes in parental attitudes as the types of information about HPV and the HPV vaccine continue to flood the literature. The information about the role of oncogenic HPV in more than cervical cancer is certainly evolving. We are just beginning to grasp the prevention implications of the HPV vaccine in the prevention of anal, oropharengeal, and a proportion of vulvovaginal, and penile cancers. The recent approval of the vaccine in young men may have an impact on decreasing condyloma transmission and having an impact on the rise of anal dysplasia/cancer in the male having sex with male population. As the cervical screening strategy moves toward primary HPV testing, this will also enhance education of the population. Although cost did not emerge as a significant barrier, as the vaccine prices continue to fall, it will be fascinating to see the impact on parental attitudes and behaviour. As public health looks at successful population-based prevention strategies, it will be interesting to look at parental attitudes toward passive consent versus active consent in school-based vaccination programs. Time will provide information on how durable the vaccine is and long-term sequelae; whether this will influence parental attitudes remains to be seen. 

In terms of future implications for policy, when the goal is to preserve the health of the population, certainly the passive consent approach, whether it is for vaccination or cervical screening, seems to be showing profound benefits. There is preliminary data that shows women who are vaccinated have less need for cervical precancer procedures like biopsies and treatment, however, how this will impact guidelines and availability of such services in the future remains to be seen.

## 6. Conclusion

Initial awareness of the virus and the ability of the virus to cause cancer have increased in the time period under study. However, awareness of the vaccine, intent to vaccinate, rates of vaccination rose during the initial introduction of the HPV vaccine but have fallen in subsequent years. Surveys have confirmed that parents want more knowledge and reassurance from their physicians that the HPV vaccine is safe for their children to receive. Policy programs, aimed at increasing HPV vaccination rates as part of an overall HPV strategy to reduce the incidents of cancers and infections caused by the virus, will need to heed the parents' concerns and information needs to be effective.

## Figures and Tables

**Table 1:** Included studies (by research question and focus).

First author	Publication date	Year survey distributed	Where study completed	Number of parents	Percent of mothers	Research question (purpose or intent)	Knowledge focus	Behaviour focus	Attitude focus
Allen	2010	2007	USA	451	Not specified	Determine factors influencing parental decisions regarding HPV vaccination in young girls	Yes	Yes	Yes
Askelson	2010	2010	USA	217	100%	To investigate the influences of mothers' intentions to vaccinate their 9- to 15-year old daughters against HPV	Yes	No	Yes
Barnack	2010	2006	USA	100	76%	Examined potential predictors of parents' willingness to vaccinate their children for HPV and physicians' intentions to encourage parents to vaccinate their children	No	No	Yes
Bernat	2009	2007	USA	1504	72.50%	Assess support for the HPV vaccine among a representative sample of parents across Minnesota	Yes	No	Yes
Brabin	2006	2005	GB/EU	317	Not specified	Assess perceptions and attitudes to HPV vaccination as determinants of acceptance of HPV policies among a representative sample of parents of young adolescents living in Manchester	Yes	No	Yes
Breitkopf	2009	2007	Asia	139	82.7%	Examine parents' perceptions of the role of mothers, fathers, and daughters in the decision to have the daughter receive the HPV vaccine; also examined perceived concordance between spouses and between parents and their daughters with regard to vaccine acceptance	Yes	No	Yes
Brewer	2011	2007	USA	650	94%	(1) Characterize HPV vaccine initiation by a racially diverse sample of adolescent girls from both rural and urban areas with elevated rates of cervical cancer; (2) identify reasons for low HPV vaccine initiation rates using a longitudinal study design	Yes	Yes	Yes
Brown	2010	2008	USA	307	100%	(a) Hypothesize that consumers would have clear preferences over several features of HPV vaccines, favouring cervical cancer protection above all other features; (b) postulate that the estimated value of consumer benefits would exceed the current retail prices of HPV vaccines given the positive and increasing demand for HPV vaccines; (c) hypothesize that total uptake of HPV vaccines would increase when a 2nd vaccine was added to the US	Yes	No	Yes
Cates	2010	2008	USA	696	80.6%	Examined characteristics of parents, their adolescent daughters, and households as potential correlates of HPV vaccine awareness and information sources; associations of information sources with HPV vaccine initiation	Yes	Yes	No
Chan	2007	2006	Asia	170	100%	Studied the utility of an information pamphlet on HPV vaccine in improving acceptance of HPV vaccination for their daughters among the study subjects	Yes	No	Yes
Chow	2010	2008	Asia	1617	100%	Determine attitudes and knowledge levels around cervical cancer and HPV vaccination amongst both physicians and mothers in Asia	Yes	Yes	Yes
Constantine	2006	2006	USA	522	73%	(1) Levels of parental acceptance of HPV vaccination for adolescent and preadolescent daughters; (2) potential race/ethnicity and other subgroup disparities in acceptance rates; (3) parents' reasons for acceptance or nonacceptance	No	No	Yes
Dahlstrom	2009	2007	GB/EU	13946	58%	Examine Swedish parents' perceptions and concerns about HPV vaccination, their willingness to vaccinate their children against HPV when the vaccine is free or not and correlates of acceptability of the new HPV vaccine	Yes	No	Yes
Davis	2004	2003	USA	506	89%	Ascertain parental perception and knowledge regarding HPV and to determine predictors of parental acceptance of a prophylactic HPV vaccine for their 10- to 15-year-old adolescents	Yes	No	Yes
de Visser	2008	2008	GB/EU	353	Not specified	Identify correlates of parents' anticipated uptake of HPV vaccination for their sons and daughters	Yes	No	Yes
Dempsey	2005	2007	USA	411	84%	To measure parental acceptability of HPV vaccines—to look at the effects of HPV information on the parental acceptance of HPV vaccination	Yes	No	Yes
Dempsey	2009	2005	USA	52	100%	To compare the reasons why mothers do or do not have their adolescent daughters vaccinated against HPV	Yes	Yes	Yes
Dinh	2007	2005	Asia	181	100%	Describe general attitudes toward vaccination and toward an HPV vaccine, in particular, among female caregivers of young women, also investigated potential cultural factors that may influence HPV vaccination uptake	Yes	No	Yes
Dursun P.	2009	2007	Turkey (multiple cities)	1427	100%	To measure the basal knowledge Turkish women have about HPV and their acceptance of the HPV vaccine for themselves and their children, using a national sample of Turkish women	Yes	No	Yes
Fang	2010	2007	USA	1383	52.30%	To report on acceptability of the HPV vaccine among a national sample of adults with female children in the household and to investigate health behaviour correlates of vaccine acceptability	Yes	No	Yes
Ferris	2010	2008	USA	325	87%	Determine factors that influence parental acceptance of a mandatory HPV vaccination program	Yes	Yes	Yes
Gerend	2009	2008	USA	82	95%	Examined parents' knowledge and beliefs about HPV vaccination, as well as correlates of HPV vaccine uptake and intentions to vaccinate a daughter/son in the future	Yes	Yes	Yes
Gillespie	2011	2011	USA	81	86%	To evaluate HPV vaccine acceptance among parents and guardians of children aged 0–10 years	Yes	No	Yes
Gottlieb	2009	2007	USA	889	93%	Assess HPV vaccine uptake by adolescent girls, their parents' intentions for them to be vaccinated, and potential barriers to their vaccination in an area with elevated cervical cancer rates	Yes	Yes	Yes
Guerry	2011	2007	USA	509	86%	To determine vaccine uptake among adolescent girls, parents' intentions to vaccinate their daughters and barriers and facilitators of vaccination in a population at elevated risk for cervical cancer	Yes	Yes	Yes
Hausdorf	2007	2004	NZ/AU	2165	Not specified	Parents' willingness to vaccinate their children against HPV and impact of potential barriers to vaccination	Yes	No	Yes
Horn	2009	2008	USA	325	88.60%	Determine parents' opinions about HPV vaccine mandates to more effectively implement a universal HPV-vaccination program	Yes	Yes	Yes
Hughes	2009	2007	USA	889	94%	Examine whether HPV and HPV vaccine awareness, knowledge, and use of information sources differ by caregivers' sex, race, age, education, income, and rural/urban residence; chose to examine variables associated with cervical cancer disease burden	Yes	Yes	No
Ilter	2010	2009	GB/EU	131	100%	To examine Muslim Turkish women's knowledge about cervical cancer screening (Pap smear) test, HPV, HPV vaccine, and their attitude toward vaccination to themselves and their daughters	No	No	Yes
Kahn	2009	2007	USA	7207	100%	To examine mothers' intention to vaccinate their daughters and themselves against HPV and to determine which demographic, behavioural, and attitudinal factors were associated with intention to vaccinate daughters	No	No	Yes
Kang	2010	2009	Asia	667	100%	To examine the attitudes, intentions, and perceived barriers to HPV vaccination among Korean high school girls and their mothers	Yes	No	Yes
Lenselink	2007	2007	GB/EU	356	91%	Assess whether Dutch parents agree to vaccinate their children against HPV infections, which factors influence their decisions and to study their knowledge about HPV, cervical cancer and HPV vaccination	Yes	No	Yes
Marlow	2008	2006	GB/EU	296	100%	Willingness to have their daughters vaccinated	No	No	Yes
Marlow	2007	2007	GB/EU	684	100%	To examine the association between general vaccine attitudes, trust in doctors and the government, past experience with vaccination, and acceptance of HPV vaccination	No	No	Yes
Marlow	2009	2006	GB/EU	332	100%	To examine the prevalence and predictors of the belief that HPV vaccination will result in “risk compensation,” that is, will increase risky sexual behaviour	Yes	No	Yes
Marshall	2007	2006	NZ/AU	601	Not specified	Assess community (adult and parental) attitudes in both men and women to the introduction of HPV vaccines in metropolitan and rural South Australia	No	No	Yes
Mortensen	2010	2010	GB/EU	450	73%	Assess parental attitudes towards male HPV vaccination in terms of their acceptance, refusal, or doubts, and who they relied on for information	No	Yes	Yes
Ogilvie	2010	2009	Canada	2025	84.90%	Assess the level of uptake of the first dose of the HPV vaccine and to determine the factors associated with receipt of the HPV vaccine	Yes	Yes	Yes
Ogilvie	2008	2007	Canada	2083	73.50%	Ascertain parental intentions to vaccinate their sons against HPV in Canada and to determine factors that predict parental intention to vaccinate their sons against HPV	Yes	No	Yes
Pelucchi	2010	2008	GB/EU	2331	52.60%	Provide basic data necessary for the development of adequate training programs for health professionals	Yes	No	Yes
Podolsky	2009	2009	USA	308	100%	Comparing two populations to gain insight into the potential impact of differences such as vaccine availability, media attention, attitudes about vaccines in general, and knowledge about HPV and vaccine acceptability	Yes	No	Yes
Rand	2011	2008	USA	382	100%	Factors influencing acceptance of vaccine: perceived susceptibility to HPV; benefits of vaccination, safety concerns; parents satisfaction with communication of vaccine	Yes	Yes	Yes
Reiter	2010	2008	USA	617	83%	Assessing vaccine initiation for HPV	No	Yes	Yes
Reiter	2010	2010	USA	406	100%	Acceptability after FDA approval for sons	Yes	No	Yes
Reiter	2009	2007	USA	889	94%	Identify parent beliefs associated with HPV vaccine initiation; determine if associations differed by race and urban/rural status	No	Yes	Yes
Reiter	2011	2011	USA	647	Not specified	Assess correlates of uptake of 3 vaccines (tetanus booster, meningococcal, and HPV vaccines) recommended for adolescent females	No	Yes	No
Rose	2010	2008	NZ/AU	769	94.3%	To describe parents' preferences on where their daughters receive the HPV vaccine, at what age, and their information needs	No	No	Yes
Rosenthal	2008	2007	USA	153	89%	Examine relationships of demographics, parenting, and vaccine attitudes with the acceptance of HPV vaccine or to the intent to vaccinate in the next 12 months	No	Yes	Yes
Toffolon-Weiss	2008	2007	USA	80	80%	To describe Alaska native-parents knowledge of and attitudes towards cervical cancer, HPV, and HPV vaccine	Yes	No	No
Tozzi	2009	2007	GB/EU	807	100%	(1) Assess parents' knowledge about HPV and HPV vaccination and their willingness to have their daughters immunized; (2) to investigate the roles of the different medical specialists in the immunization strategy as perceived by parents	Yes	No	Yes
Woodhall	2007	2005	GB/EU	727	70%	To examine acceptance of HPV vaccination by adolescents and their parents	Yes	No	Yes
Yeganeh	2010	2008	USA	95	100%	Examine factors associated with parental consent for HPV vaccination one year after vaccine implementation as well as parental support for an HPV vaccine mandate for middle-school-age children	Yes	Yes	Yes
Ziarnowski	2009	2007	USA	889	94%	Examined the role of anticipated regret in caregivers' HPV vaccination decisions as well as potential antecedents of anticipated regret	No	Yes	Yes

**Table 2:** Summary of trends prior to 2007 and after 2007.

	Number of studies	Range values 2007 or earlier		Number of studies	Range values 2008 or later	
	2007 or earlier	min %	max %	2008 or later	min %	max %
Heard of HPV	12	59.0	59.5	7	64.7	93.0
Heard of HPV vaccine	10	14.0	58.7	5	47.0	64.5
Understood connection between HPV and Cervical Cancer	4	8.0	70.0	1	53.4	91.0
Parent intends to vaccinate child	22	54.0	86.0	8	47.0	79.5
Child is vaccinated HPV	NA	NA		17	24.9	84.0
